# Neural Activity Patterns in the Human Brain Reflect Tactile Stickiness Perception

**DOI:** 10.3389/fnhum.2017.00445

**Published:** 2017-09-04

**Authors:** Junsuk Kim, Jiwon Yeon, Jaekyun Ryu, Jang-Yeon Park, Soon-Cheol Chung, Sung-Phil Kim

**Affiliations:** ^1^Department of Human Perception, Cognition and Action, Max Planck Institute for Biological Cybernetics Tübingen, Germany; ^2^Department of Brain and Cognitive Engineering, Korea University Seoul, South Korea; ^3^School of Psychology, Georgia Institute of Technology Atlanta, GA, United States; ^4^Center for Neuroscience Imaging Research, Institute for Basic Science Suwon, South Korea; ^5^Department of Biomedical Engineering, Sungkyunkwan University Suwon, South Korea; ^6^School of Biomedical Engineering, Konkuk University Chungju, South Korea; ^7^Department of Human Factors Engineering, Ulsan National Institute of Science and Technology Ulsan, South Korea

**Keywords:** fMRI, MVPA, tactile stickiness, neural correlates, categorization, perception

## Abstract

Our previous human fMRI study found brain activations correlated with tactile stickiness perception using the uni-variate general linear model (GLM) (Yeon et al., 2017). Here, we conducted an in-depth investigation on neural correlates of sticky sensations by employing a multivoxel pattern analysis (MVPA) on the same dataset. In particular, we statistically compared multi-variate neural activities in response to the three groups of sticky stimuli: A supra-threshold group including a set of sticky stimuli that evoked vivid sticky perception; an infra-threshold group including another set of sticky stimuli that barely evoked sticky perception; and a sham group including acrylic stimuli with no physically sticky property. Searchlight MVPAs were performed to search for local activity patterns carrying neural information of stickiness perception. Similar to the uni-variate GLM results, significant multi-variate neural activity patterns were identified in postcentral gyrus, subcortical (basal ganglia and thalamus), and insula areas (insula and adjacent areas). Moreover, MVPAs revealed that activity patterns in posterior parietal cortex discriminated the perceptual intensities of stickiness, which was not present in the uni-variate analysis. Next, we applied a principal component analysis (PCA) to the voxel response patterns within identified clusters so as to find low-dimensional neural representations of stickiness intensities. Follow-up clustering analyses clearly showed separate neural grouping configurations between the Supra- and Infra-threshold groups. Interestingly, this neural categorization was in line with the perceptual grouping pattern obtained from the psychophysical data. Our findings thus suggest that different stickiness intensities would elicit distinct neural activity patterns in the human brain and may provide a neural basis for the perception and categorization of tactile stickiness.

## Introduction

The perception of surface texture is of great importance to interact with the environment in our daily life. Since most natural objects are characterized not only by its shape but also by its surface texture, a precise tactile perception with one's hands plays an essential role in object recognition and manipulation (Klatzky et al., 1987; Johansson and Flanagan, 2009). To date, a number of psychophysical studies have investigated how humans perceive surface texture properties and proposed fundamental dimensions of tactile perception (Hollins et al., 2000; Yoshioka et al., 2007; Bensmaia, 2016). In general, there are four fundamental dimensions in tactile perception (i.e., roughness/smoothness, hardness/softness, stickiness/slipperiness, and warmth/coolness) and the surface texture perception is mainly determined by the tactile dimensions of roughness, hardness, and stickiness (Bensmaia, 2016). The neural basis of texture perception has been explored based on two distinct mechanisms mediated by different cutaneous mechanoreceptors (Bensmaia, 2016). The slowly adapting type 1 (SA1) afferents mediate a spatial mechanism, and rapidly adapting (RA) and Pacinian (PC) afferents mediate a temporal mechanism of surface texture perception. These texture-related afferent signals lead to the neural responses in the primary somatosensory cortex (S1). Many neuroimaging studies have explored neural mechanisms for the texture perception in the human brain (for a review, see Bensmaia, 2016). For instance, in a functional magnetic resonance imaging (fMRI) study by Reed et al. (2004), participants explored various tactile objects along multiple dimensions (e.g., hardness, roughness) and conducted a tactile object recognition task during the brain image acquisition. Their results demonstrated a primary role of somatosensory association areas in the tactile stimulus recognition. More specifically, for the roughness dimension, previous electrophysiological recording and lesion studies have shown that the tactile roughness information is encoded in S1 (Randolph and Semmes, 1974; Chapman et al., 2002) and S2 (Murray and Mishkin, 1984; Pruett et al., 2000). For the hardness dimension, several fMRI studies suggested that neural activities in the postcentral gyrus and parietal operculum play a role in sensing tactile hardness during object recognition (Servos et al., 2001; Reed et al., 2004). However, compared with other dimensions, relatively less attention has been devoted to the sticky sensation in spite of its significance for surface texture perception (Bensmaia, 2016).

Most previous studies have investigated stickiness perception from the view of the opposite of slipperiness perception (i.e., non-slipperiness; van Kuilenburg et al., 2015; Bensmaia, 2016). In other words, stickiness perception has been studied by examining the kinetic friction between surface texture and a fingertip, occurring when the finger slides over the surface. For example, Smith and Scott showed that magnitude estimates of stickiness perception are closely related to a ratio between the force exerted perpendicular to the surface to that parallel to the plane of the surface (Smith and Scott, 1996). Their findings indicated a close relationship between the tangential force and tactile stickiness perception, as participants in that study applied a similar level of perpendicular forces to the surface consistently during the experiment. Liu and colleagues also measured the kinetic friction between the finger and the surface texture of aluminum samples and found a correlation between the friction levels with the perceived grippy-slippery ratings (Liu et al., 2008). These previous findings undoubtedly demonstrated the contribution of tangential forces to the stickiness perception (Smith and Scott, 1996; Liu et al., 2008; Bensmaia, 2016). However, we perceive sticky sensations evoked not only by tangential forces but also by perpendicular adhesive forces from our everyday items (e.g., post-it notes and tapes). Nonetheless, little is known about tactile stickiness perception when the skin is pulled from the adhesive material.

Only a few studies have so far explored tactile stickiness perception evoked by the pull-off force (Zigler, 1923; Yamaoka et al., 2008). Yamaoka and colleagues examined the temporal dynamics of sticky sensation using a vacuum adhesive pressure device and found interactions between the applied perpendicular force and the contact surface area on sticky perception (Yamaoka et al., 2008). Another psychological study showed that humans perceive a sticky sensation at the moment when the skin begins detaching from the object surface, suggesting sticky sensation is closely related to the skin stretch (Zigler, 1923). As this skin stretch is known to be associated with slowly adapting type 2 (SA2) afferents ending in Ruffini corpuscles (Johansson and Flanagan, 2009), SA2 afferents have been considered as mechanoreceptive fibers underlying stickiness perception. In spite of these neuropsychological findings, it remains largely unknown how sticky stimulations are perceived in the human brain.

In our previous fMRI study, we successfully found brain regions exhibiting correlations of blood oxygen level-dependent (BOLD) signals with perceived tactile stickiness using conventional uni-variate analyses and an in-house sticky stimulus set (Yeon et al., 2017). Based on the psychophysical experimental results on our stimulus set, the sticky stimuli were divided into three disjoint groups in terms of perceptual intensity: Supra-threshold (sticky stimuli that can consistently evoke sticky sensation), Infra-threshold (sticky stimuli that barely evoke sticky sensation), and Sham (tactile stimuli that contain no sticky property) groups. Using a general linear model (GLM) analysis, we statistically evaluated the BOLD responses of three different contrasts: (a) Supra-threshold against Sham stimuli, (b) Supra-threshold against Infra-threshold stimuli, and (c) Infra-threshold against Sham stimuli. It was in the case (a) when we observed significant neural activations in the contralateral primary somatosensory area (S1) and ipsilateral dorsolateral prefrontal cortex (DLPFC), while in the case (b) we observed significant neural activations in subcortical regions including putamen and thalamus. In the case of (c), no significant neural activation was observed. The results of this previous study have revealed human brain responses to tactile perception of sticky stimuli for the first time, yet some aspects of stickiness perception still remain unexplored. For example, we observed distinct behavioral responses to the different stickiness intensities within the Supra-threshold group, but uni-variate GLM analyses did not identify neural correlates of the perceptual sensitivity to stickiness intensity. To the best of our knowledge, this is the first attempt to explore a neural mechanism of the tactile stickiness perception via the neural activation pattern analysis.

To overcome the limitation of uni-variate analyses, the present study examined neural activation patterns dependent on the tactile stickiness perception using the same dataset in our previous study. Multivoxel pattern analysis (MVPA) was employed to decode voxel response patterns evoked by different sticky stimuli. In particular, we searched for the brain regions exhibiting neural activity patterns representing stickiness perception using searchlight analysis (Kriegeskorte et al., 2006). Since MVPA characterizes spatial neural activity patterns encoded in the human brain (for a review, see Tong and Pratte, 2012), one could expect novel findings about stickiness information processing in addition to the outcomes from uni-variate analyses. Several fMRI studies have already utilized MVPA to explore tactile information processing as a complementary method to GLM (Hartmann et al., 2008; Kim et al., 2015b). Once we identified neural activations that would allow discrimination between different stickiness percepts, we applied a dimensionality reduction technique to find a low-dimensional neural representation of a high-dimensional multivoxel patterns. Dimensionality reduction enables visualization of high-dimensional data such as multivoxel patterns and provides us an adequate depiction for the stickiness intensity distributions embedded in the voxel response patterns (Walther et al., 2016). We employed principal component analysis (PCA) as it has been one of the widely used dimensionality reduction techniques in neuroimaging (Abdi et al., 2009; Shinkareva et al., 2012; Brouwer and Heeger, 2013).

## Materials and methods

### Participants and ethics approval

Nine healthy volunteers (5 females, average 24.6 ± 2.47 years old, age range: 20–29 years old) with no contraindications against MR investigations and no history of neurological disorders participated in the experiment. All participants were right-handed and had no deficits in tactile processing. Experimental procedures were approved by the ethical committee of Ulsan National Institute of Science and Technology (UNISTIRB-15-16-A) and the study was conducted in accordance with the Declaration of Helsinki. Informed consent was obtained from all participants.

### Tactile stimuli and experimental design

To elicit different levels of sticky sensation, we used custom-made silicone-based sticky stimuli (polydimethylsiloxane; PDMS; Yi et al., 2014). The materials were created by mixing the fast catalysts (CA-5275, GT Products Inc., TX, USA) into the liquid silicone (GT5727, GT Products Inc., TX, USA) with different ratios, i.e., 5, 6, 7, 8, 10, and 30%. Because different mixing ratios produce distinct intensities of the stickiness, 6 different sticky stimuli were prepared for the fMRI experiment. Note that the lower the catalyst ratio was, the less the silicone was hardened, which made the material stickier. In addition, a sham stimulus without a sticky property made of acryl was prepared to present a non-sticky stimulation. Hence, a total of 7 intensities of sticky stimulus were used for the fMRI experiment. These stimuli were formed into a single cylinder shape with a 35-mm diameter and a 5-mm height and each stimulus was attached on an acrylic plate sized 80 × 50 mm^2^.

To minimize potential confounding factors due to finger-movement variation, participants performed two training sessions outside the MR room in advance of the main experiments. In the first training session, participants were trained to exert a pressure force of 1 N consistently on a right index fingertip. Since the perception of stickiness is closely associated with the force exerted perpendicular to the surface (Bensmaia, 2016), the equalization of exerted forces is necessary to evoke a similar level of sticky sensation across the participants as well as the trials. When the force was applied on a pressure sensor (A201-100, FlexiForce, MA, USA), the values of the pressure were displayed immediately on the monitor as a bar graph. With this visual feedback, participants were able to adjust the amount of the exerted pressure in real time. Moreover, participants performed the second training session to regularize the right index finger movements. They practiced “Attaching,” “Detaching,” and “Resting” finger postures following the instructions of experimenter (Yeon et al., 2017).

Prior to the fMRI experiment, two psychophysical experiments were carried out to investigate a relationship between perceived stickiness and physical stimuli. To estimate an absolute threshold of the tactile perception of stickiness, we employed a classical psychophysical method (method of constant stimuli). Moreover, we utilized an adaptive psychophysical method (magnitude estimation) to quantitatively measure the perceived intensity of stickiness sensation (see Yeon et al., 2017 for more details of behavioral experiments).

During the functional image acquisition, participants laid supine in the MR scanner with their right arm comfortably placed along the magnet bore. They wore a headset to listen to auditory instructions and an eye patch to block visual information. An experimenter was positioned at the entrance of the magnet bore where one could easily reach out to participants and placed the stimulus plate on the MR table for each trial in a consistent manner. The experiment consisted of two fMRI runs, each started with a 6 s of baseline period followed by a series of 70 trials (7 stickiness intensities × 10 repetitions). Each trial was composed of 3 distinct finger movements: “Attaching” the right index fingertip to a given sticky material when participants heard the verbal instruction and maintaining the pose for 3 s, “Detaching” the right index fingertip from the stimulus as soon as they heard a short beep sound, and “Resting” the right index fingertip on the MR table after the stimulus plate was removed by the experimenter for 15 s until the next trial. The duration of each trial was thus 18 s and each run lasted 21 min 6 s. The presentation order was randomized and no specific behavioral response was recorded in each trial.

### Data acquisition and preprocessing

Neuroimaging data were acquired using a 3-T MRI system (Magnetom TrioTim, Siemens Medical Systems, Erlangen, Germany) equipped with a standard 12-channel head coil. Anatomical images were obtained using a T_1_-weighted 3D MPRAGE sequence with repetition time (TR) = 1,900 ms, echo time (TE) = 2.48 ms, flip angle = 9°, field of view (FOV) = 200 mm, and spatial resolution = 0.8 × 0.8 × 1 mm^3^. 47 axial functional images were obtained BOLD sensitive gradient-echo-based echo planar imaging (GE-EPI) sequence with TR = 3,000 ms, TE = 30 ms, flip angle = 90°, FOV = 192 mm, slice thickness = 3 mm, and in-plane resolution = 2 × 2 mm^2^. The coverage of functional images was the whole cerebrum. Standard preprocessing of the fMRI data was performed using SPM8 (Wellcome Department of Imaging Neuroscience, UCL, London, UK) and a high-pass filter of 128 s was used to remove low frequency noise. The EPI data were corrected for slice-timing differences, realigned for motion correction, co-registered to the individual T_1_-weighted images, normalized to the Montreal Neurological Institute (MNI) space, and no spatial smoothing was applied for MVPA.

### fMRI data analysis

#### Searchlight MVPA

In this study, we applied multi-variate analytic approaches on the same fMRI data set that were used in our previous uni-variate GLM study (Yeon et al., 2017). Particularly, we searched for local neural activity patterns that would allow differentiating between stickiness perceptual groups using searchlight MVPA (Kriegeskorte et al., 2006). Searchlight techniques have been successfully utilized to decode multi-variate neural activity patterns into tactile information such as vibrotactile frequency (Kim et al., 2015b) and tactile roughness information (Kim et al., 2015a). As input features to searchlight analysis, we extracted parameter estimates of the voxel response to each stickiness intensity using a GLM (implemented in SPM8). Since sticky sensations are evoked when the skin is stretched by sticky surfaces (Yamaoka et al., 2008), we determined the moment of “Detaching” the fingertip from the sticky surface as a stimulus onset. Standard predictors were made by the convolution of stimulus onsets with the standard model of the hemodynamic response function. Overall, a total of 140 event-related regressors (7 stimulus intensities × 10 repetitions × 2 fMRI runs) were used to predict the voxel response of each participant. Obtained parameter estimates were used as input to a searchlight MVPA (implemented in the Searchmight Toolbox; Pereira and Botvinick, 2011). In our analysis, a searchlight with a 7 × 7 × 7 cube was constructed to run through the whole brain volume.

Our initial goal of searchlight analysis was to seek brain areas in which local activation patterns allowed decoding of 6-class stickiness intensities (5, 6, 7, 8, 10, and 30% catalyst ratio stimuli). Yet, we did not find any brain region showing significant decoding performance, i.e., no significant cluster was found at *p* < 0.001 (uncorrected). We had speculated that the neural activity patterns evoked by 6 sticky stimuli could not be distinguishable due to an insufficient spatial resolution of fMRI data obtained in this study. Therefore, instead of the 6-class decoding, we performed binary classifications in each searchlight cube to predict one of the two sticky stimuli, which could represent each perceptual group. According to behavioral results (Yeon et al., 2017), the mean absolute threshold of sticky sensation was a 7.47 ± 1.3% catalyst ratio. In other words, a presented sticky stimulus was perceived as non-sticky if the sticky material was made of catalyst ratio higher than 7.47%, even though it contained sticky property. Based on this finding, we divided 7 sticky stimuli into 3 groups: the Supra-threshold group consisting of 5, 6, and 7% catalyst ratio stimuli, the Infra-threshold group consisting of 8, 10, and 30% catalyst ratio stimuli, and the Sham group of the acrylic sham stimulus. To seek brain regions containing neural activity patterns related to tactile stickiness perception, we performed 3 searchlight analyses: Decoding multivoxel features into one of the two sticky stimuli between (1) 5% and 30% stimuli representing the Supra- and Infra-threshold groups, respectively, (2) 5% and sham representing the Supra-threshold and Sham groups, respectively, and (3) 30% and sham representing the Infra-threshold and Sham groups, respectively. As for the statistical classifier, we used a Gaussian Naïve Bayes (GNB) classifier that has been one of the widely used classifiers for the searchlight analysis (Mitchell et al., 2004; Pereira and Botvinick, 2011). Moreover, a GNB classifier provides a reasonable solution for quick mapping and voxel selection in consideration of computational loads (Pereira and Botvinick, 2011). As described in the previous paragraph, we used the parameter estimates vectors obtained from the GLM modeling as classifier inputs. The dimensionality of the parameter estimates vector was equal to the number of voxels in a searchlight (i.e., 343). In the cross-validation, 2-fold refers to the 2 different data groups for training and testing: Fold “1” indicates the data obtained from the first fMRI run, and fold “2” indicates the data obtained from the second fMRI run. For the decoding analyses, 20 parameter estimates vectors (2 stimulus intensities × 10 repetitions) from the first fMRI run were used as a training set. Then, the trained classifier classified another 20 parameter estimates vectors from the second fMRI run in the test phase. This testing procedure was repeated again by swapping the folds for training and testing. The resulting decoding accuracy from both cross-validations was averaged and assigned to the center voxel in the searchlight. The chance-level accuracy (0.5 in this case) was then subtracted from the values stored in each voxel to represent deviations from chance. Accuracy maps obtained from individual participants were used for a subsequent random-effects group analysis, which was implemented as one-sample *t*-test against 0 to identify above-chance decoding accuracy for every voxel.

To correct a multiple comparisons problem, we estimated an empirical cluster size threshold for a group of participants in the searchlight accuracy maps. Following a randomization procedure described by Oosterhof et al. (2010), the size of the clusters obtained in the group analysis was compared to a reference distribution of clusters obtained by chance. Under the no-effect condition, the sign of the searchlight decoding accuracy values (positive or negative after subtraction of a chance level) would be randomly assigned with a probability of 50%. To identify how large clusters would be in this null hypothesis, we sampled the searchlight accuracy maps and randomly flipped the sign of the maps of a random number of participants. These maps were considered as one group sample under the null hypothesis and a random-effect analysis on these maps calculated the largest size of the cluster. The distribution of the largest cluster sizes under the null hypothesis was obtained from 1,000 repetitions. In this study, we reported clusters as significant if its size was in the 5% of the upper tail of the null distribution, i.e., *p* < 0.05 corrected for multiple comparisons via the cluster size.

Behavioral results of magnitude estimation in our previous study suggested that the Supra-threshold group could be further divided into two subgroups (Yeon et al., 2017). Especially, the stimulus with the 7% catalyst ratio could be differently perceived from the 5 and 6% stimuli. However, the perceived sticky intensity was not significantly different between the 5 and 6% stimuli. To find a neural correlate of this behavioral observation, we performed an additional 3-class searchlight MVPA. In this analysis, a GNB classifier was built to discriminate 3 categories of stickiness intensity (i.e., 5 vs. 7 vs. 30% stimuli). As a training set, 30 parameter estimates vectors (3 stimulus intensities × 10 repetitions) from the first fMRI run were used. Then, the classifier discriminated another 30 parameter estimates vectors from the second fMRI run in the test phase. Other decoding analysis steps were identical to the aforementioned searchlight analysis except for the chance level (33.3% here). Additionally, we computed the confusion matrices for each searchlight analysis. Since the searchlight analyses focused only on the correct classification rates (i.e., decoding accuracies), it is worthwhile to investigate the mis-classification pattern as a complementary analysis.

#### Dimensionality reduction analysis

Once we found significant clusters that carry information of stickiness perception, we attempted to visualize a spatial distribution of neural activity patterns evoked by 7 levels of stickiness intensity (6 stickiness levels and sham stimulus). We assumed that the visual depiction of voxel response patterns based on distance metrics could demonstrate the degree of separation between stickiness intensities in a feature space. To this end, we extracted the multi-voxel patterns, which were the parameter estimates from the GLM in response to each stickiness intensity, in the clusters identified by each of the searchlight analyses. For instance, in the case of the searchlight analysis discriminating 5 vs. 30% stimuli, significant clusters were found in postcentral gyrus and thalamus, containing a total of 50 voxels. Then, we extracted the parameter estimates of the GLM (i.e., beta values) for each voxel in response to each stimulus (i.e., 6 stickiness levels and sham) for each participant. It generated a 7 × 50 multivoxel pattern matrix per participant. Then, using a weighted average procedure (*Structuration des Tableaux A Trois Indices de la Statistique*; STATIS) described by Shinkareva et al. (2012), we created cross-product matrices representing the similarity between 7 stickiness intensities for each participant and combined these matrices into a compromise matrix. PCA was applied to the compromise matrix to extract the first two principal components. Therefore, although the clusters identified as significant for the decoding of 2 or 3 stickiness levels were of interest, the dimensionality reduction analysis formed a 2-dimensional neural representation for all 7 stickiness levels. This procedure of visualization was repeated for every searchlight analysis where the entire procedure was the same except for the target clusters of the voxels between the searchlight analyses.

To investigate the correspondence between neural and behavioral responses of stickiness perception, we compared grouping patterns of neural and behavioral representations of stickiness intensities. As for the behavioral responses of sticky stimuli, we extracted magnitude estimation responses of each stickiness intensity and normalized them to z-scores (see Yeon et al., 2017 for more details of behavioral experiments). These values were averaged across participants and mapped into a 2-dimensional space. Next, we utilized a *k*-means clustering analysis, which partitioned a given data set into a certain number of groups that clustered around each of *k* centroids. Clusters were formed so as to minimize the sum of point-to-cluster-centroid Euclidean distances (MacKay, 2003). Similarly, 2-dimensional neural representations of stickiness intensities obtained from PCA were categorized using the same *k*-means clustering analysis. We then directly compared neural and behavioral grouping patterns of sticky stimuli for *k* = 2 or 3.

## Results

### Searchlight MVPA

**Figure 1:**
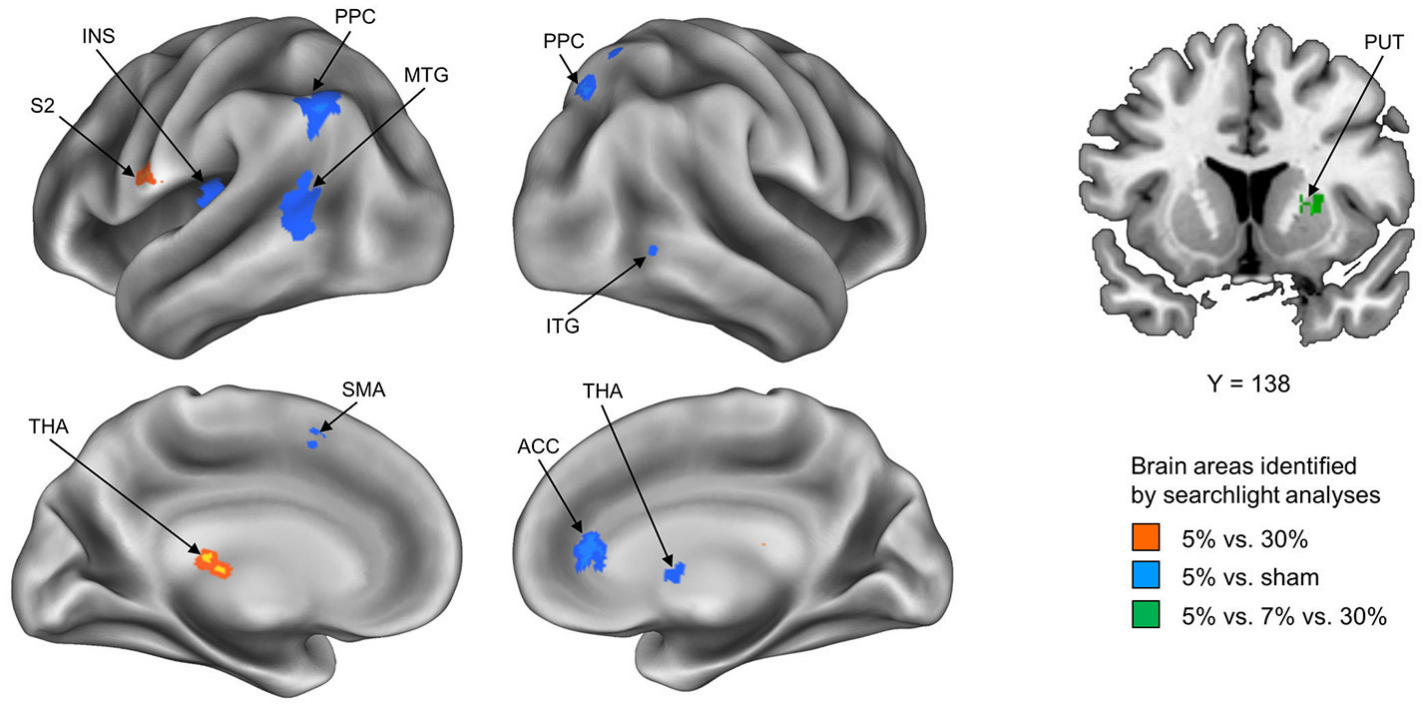
Summary of the whole-brain searchlight analyses. Three searchlight analyses identified significant brain regions exhibiting neural activity paCtterns conveying neural information of tactile stickiness perception. Each searchlight analysis discriminated between Supra- and Infra-threshold group, i.e., 5 vs. 30% (Red), Supra-threshold and Sham group, i.e., 5% vs. sham (Blue), and Two subgroup of Supra- and Infra-threshold group, i.e., 5 vs. 7 vs. 30% (Green). S2, secondary somatosensory area; THA, thalamus; PPC, posterior parietal cortex; MTG, middle temporal gyrus; INS, insula; SMA, supplementary motor area; ITG, inferior temporal gyrus; ACC, anterior cingulate cortex; PUT, putamen.

To explore how stickiness perception is represented in multivoxel patterns of BOLD signals, we performed 4 different searchlight analyses. Resulting accuracy maps for each participant were taken to a random-effects group analysis to establish commonalities. The first searchlight analysis, which decoded the 5% and 30% stimuli that represented the Supra- and Infra-threshold groups, respectively, found two significant clusters: The postcentral gyrus (including second somatosensory area; S2) and the thalamus in the contralateral hemisphere (*p* < 0.05 cluster size corrected, size > 20) (Figure 1 and Table 1). The second searchlight analysis, which decoded the 5% and sham stimuli that represented the Supra-threshold and Sham groups, respectively, found eight significant clusters: The posterior parietal cortex (PPC) in the bilateral hemisphere, the middle temporal gyrus (MTG), the insula, the supplementary motor area (SMA) in the contralateral hemisphere, and the thalamus, the inferior temporal gyrus (ITG), the anterior cingulate cortex (ACC) in the ipsilateral hemisphere (*p* < 0.05 cluster size corrected, size > 20) (Figure 1 and Table 1). The third searchlight analysis, which decoded the 30% and sham stimuli that represented the Infra-threshold and Sham groups, respectively, did not find any significant cluster. The fourth searchlight analysis, which decoded the 5%, 7%, and 30% stimuli that represented two Supra-threshold subgroups and Infra-threshold groups, respectively, found a significant cluster: The putamen in the ipsilateral hemisphere (*p* < 0.05 cluster size corrected, size > 20; Figure 1 and Table 1). These clusters remained significant after correction for multiple comparisons via the cluster size (Oosterhof et al., 2010).

**Table 1:** Identified brain regions in which the local activity patterns allowed to discriminate the stickiness perceptual groups (*p* < 0.05 cluster size corrected, size > 20).

**Regions**	**Side**	**MNI coordinates**			**Voxels**	***T***	***Z***	**Accuracy ± SE (%)**
		***X***	***y***	***Z***				
**5 vs. 30%**								
Postcentral gyrus	Left	−64	−8	14	22	9.25	4.33	67.8 ± 4.4
Thalamus	Left	−2	−32	8	28	5.77	3.53	63.4 ± 4.1
	Left	−10	−38	12		5.50	3.44	
**5% vs. sham**								
Thalamus	Right	4	0	0	37	11.39	4.66	67.3 ± 4.5
	Right	6	−8	2		5.49	3.44	
	Right	2	−2	8		5.43	3.42	
Posterior parietal cortex	Left	−44	−64	42	137	10.74	4.57	72.6 ± 4.3
	Left	−38	−62	36		6.04	3.61	
	Left	−42	−60	52		5.31	3.38	
Middle temporal gyrus	Left	−50	−60	4	138	10.20	4.48	72.7 ± 5.3
	Left	−48	−62	16		8.70	4.23	
	Left	−58	−62	10		6.87	3.83	
Inferior temporal gyrus	Right	44	−54	−6	35	8.65	4.22	73.4 ± 5.1
	Right	36	−50	−8		8.08	4.10	
Insula	Left	−38	−18	14	22	7.46	3.97	68.5 ± 4.1
Supplementary motor area	Left	−10	12	52	20	7.16	3.90	74.6 ± 4.9
Anterior cingulate cortex	Right	2	40	−4	45	6.70	3.79	70.6 ± 5.7
	Right	4	36	4		6.59	3.76	
Posterior parietal cortex	Right	28	−66	58	50	6.67	3.78	76.6 ± 4.2
	Right	32	−72	52		5.15	3.33	
	Right	18	−64	52		5.11	3.32	
**30% vs. sham**								
	No significant clusters found							
**5 vs. 7 vs. 30%**								
Putamen	Right	24	10	4	21	7.75	4.03	45.2 ± 3.3

*Cluster, size indicates N voxels; T, indicates peak t-values; Z, indicates peak z-values. Entries without brain region labels indicate sub-peak within the cluster named above. Classification performances for each cluster identified from searchlight MVPA are shown in mean decoding accuracy ± standard error*.

**Figure 2:**
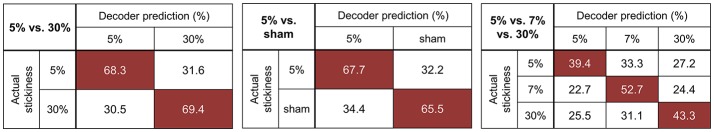
Confusion matrices for the identified clusters of each searchlight analysis. The rows of the matrix indicate the actual stickiness provided to the participants (true label) and the columns indicate the predictions by a classifier (predicted label). The cells of highest accuracy in each row are highlighted in red. The chance levels for the 5 vs. 30 and 5% vs. sham stimuli classification are 50%. As for the 5 vs. 7 vs. 30% stimuli classification, the chancel level is 33.3%.

Figure 2 illustrates the confusion matrices resulting from each searchlight analysis. The decoding accuracy on a given row *j* and column *k* of the confusion matrix indicates the proportion that a presentation of stickiness *j* was predicted as stickiness *k*. However, we could not find any specific mis-classification pattern from the confusion matrices. Figure 2 also shows the mean decoding accuracies across participants for each searchlight analysis (given in mean ± standard deviation): 68.9 ± 4.2% for the 5 vs. 30% stimuli classification; 66.7 ± 6.1% for the 5% vs. sham stimuli classification; 45.2 ± 3.3% for the 5 vs. 7 vs. 30% stimuli classification. We examined the possibility that the significant decoding accuracy obtained from the 3-class searchlight analysis (5 vs. 7 vs. 30%) was due to the high classification performance between the 5 and 30% stimuli. To this end, we tested whether the average classification performances for each diagonal entry were higher than the chance level (33.3%). As results, all category values were significantly higher than chance [all ps < 0.01, *t*_(8)_ = 4.2 for the category “5%”; *t*_(8)_ = 17.2 for the category “7%”; *t*_(8)_ = 14.7 for the category “30%”]. This test confirmed that the significance of overall accuracy in the 3-class classification was not due to the strong discrimination between the 5 and 30%.

### Dimensionality reduction analysis

**Figure 3:**
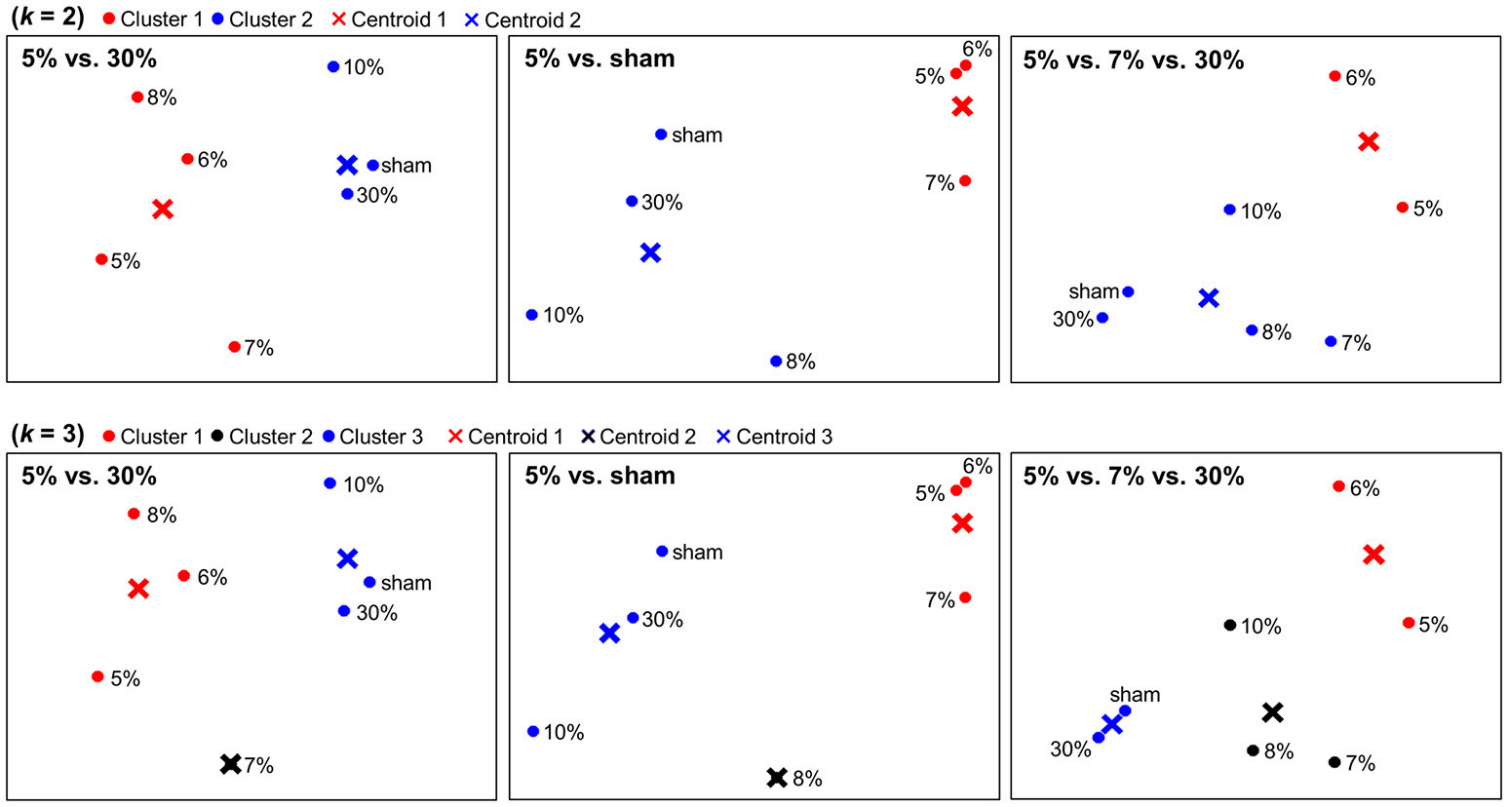
Spatial depictions of the neural representation of stickiness intensities. Using a principal component analysis (PCA), neural representations of stickiness intensities were mapped into a 2-dimensional space. A follow-up *k*-means clustering analysis revealed how the neural activity patterns of stickiness intensities were categorized together. The centroids of each group were marked in “×”. First row: Spatial configurations for neural categorizations in response to 7 stickiness intensities, when *k* = 2. Second row: Spatial configurations for neural categorizations in response to 7 stickiness intensities, when *k* = 3. Regarding associated brain regions, the first column was based on the significant clusters in postcentral gyrus and thalamus. The second column was based on the eight significant clusters including bilateral PPC, MTG, insula, SMA, thalamus, ITG, and ACC. The last column was based on the significant clusters in putamen.

The dimensionality reduction using PCA enabled us to visualize neural representations of sticky stimuli in a 2-dimensional space. Figure 3 depicts spatial configurations of stimulus categories constructed within the clusters identified from each searchlight analysis. Regarding associated brain regions, the first column was based on the significant clusters in postcentral gyrus and thalamus. The second column was based on the eight significant clusters including bilateral PPC, MTG, insula, SMA, thalamus, ITG and ACC. The last column was based on the significant clusters in putamen. PCA entails a trade-off between the comfort of a visual representation and inevitable projection artifacts, because entire variance of the data cannot be explained by only two principal components. In our application, for example, the first two principal components explained 53.8, 62.4, and 53.2% of the variance for the 5 vs. 30, 5% vs. sham, and 5 vs. 7 vs. 30% stimuli searchlight analyses, respectively. Although there was still remaining variance to deal with, we could observe clear categorical patterns in this 2-dimensional space. Other neuroimaging studies obtaining a similar level of residual variance with ours have also achieved clear categorization results (Abdi et al., 2009; Shinkareva et al., 2012).

We performed *k*-means clustering analyses to group neural representations of 7 stimuli into *k* clusters (Figure 3). When *k* = 2, within the clusters identified from the 5 vs. 30% stimuli decoding analysis, one group included the four (5, 6, 7, 8%) and the other group included the remaining three stimulus intensities (10, 30%, sham). Within the clusters identified from the 5% vs. sham stimuli decoding analysis, one group included the three (5, 6, 7%) and the other group included the remaining four stimulus intensities (8, 10, 30%, sham). In addition, within the cluster identified from the 5 vs. 7 vs. 30% stimuli decoding analysis, one group included the two (5, 6%) and the other group included the remaining five stimulus intensities (7, 8, 10, 30%, sham). When *k* = 3, within the clusters identified the 5 vs. 30% stimuli decoding analysis, one group included the three (5, 6, 8%), another group included the one (7%), and the other group included the remaining three stimulus intensities (10, 30%, sham). Within the clusters identified the 5% vs. sham stimuli decoding analysis, one group included the three (5, 6, 7%), another group included the one (8%), and the third group included the remaining three stimulus intensities (10, 30%, sham). Within the clusters identified the 5 vs. 7 vs. 30% stimuli decoding analysis, one group included the two (5, 6%), another group included the three (7, 8, 10%), and the third group included the remaining two stimulus intensities (30%, sham).

**Figure 4:**
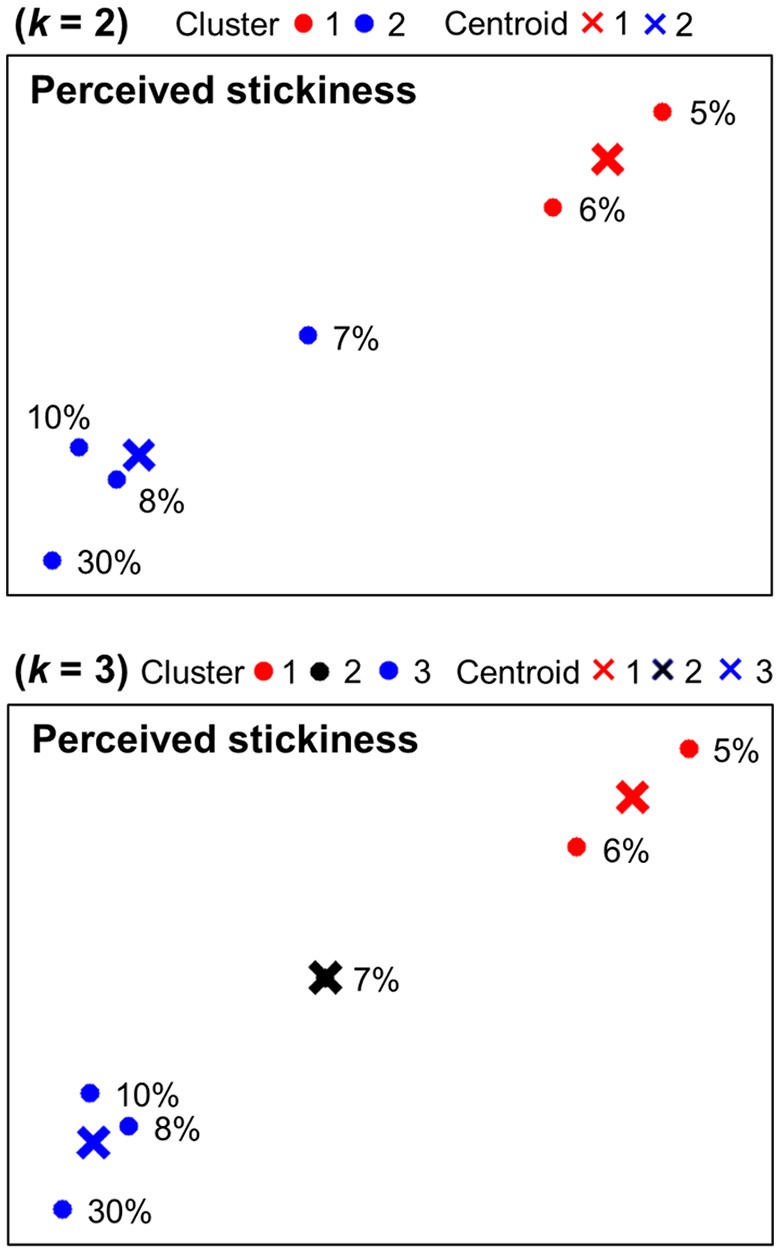
Behavioral categorization patterns of stickiness intensities. Behavioral responses of the magnitude estimation experiment were displayed in a 2-dimensional space and categorized into 2 or 3 clusters using *k*-means clustering methods. The centroids of each group were marked in “×.”

Figure 4 shows *k*-means clustering results for behavioral magnitude estimations of stickiness intensities. When *k* = 2, one group included the two (5, 6%) and the other group included the remaining four stimulus intensities (7, 8, 10, 30%). When *k* = 3, one group included the two (5, 6%), another group included the one (7%), and the third group included the remaining three stimulus intensities (8, 10, 30%).

## Discussion

### Brain activity patterns associated with stickiness perception

Our previous study identified brain regions showing significant BOLD signal changes in response to tactile stickiness perception in individual voxels using conventional GLM analyses. On top of that, the current study applied a multi-variate approach to analyze the spatially distributed neural activity patterns related to tactile stickiness perception over the whole brain. Specifically, we carried out four different searchlight MVPAs to search neural patterns underlying characteristics of tactile stickiness perception. Each of the four searchlight analyses accounted for the different aspects of stickiness perception.

We performed the first searchlight analysis, decoding the 5 vs. 30% catalyst ratio sticky stimuli, to explore brain regions carrying neural information about perceptual differences between Supra- and Infra-threshold stickiness groups. Significant clusters were identified in contralateral postcentral gyrus and thalamus. Despite both the 5 and 30% stimuli inherently contain sticky properties, participants could perceive a sticky sensation only by 5% stimulus. Therefore, this analysis seemed to reveal brain regions involved in sticky stimulus recognition. A number of previous studies have provided considerable evidence for postcentral gyrus's (S1 and S2 in the study of Beauchamp et al., 2009; S1 in the study of Martuzzi et al., 2014) and thalamus's (Tremblay et al., 1993; Vazquez et al., 2012) contributions to the perception of somatosensory inputs. In addition, several neuroimaging studies have identified the involvement of postcentral gyrus in surface texture recognition such as roughness (Osullivan et al., 1994) and hardness discrimination (Servos et al., 2001) (for a review, see Sathian, 2016). For example, an fMRI study found neural activations in the posterior parts of postcentral gyrus related to the intensity judgements of texture stimuli, suggesting the role of posterior parts of postcentral gyrus in representing surface texture intensities (Servos et al., 2001). Together with these findings, our result indicates that postcentral gyrus and thalamus may take part in sticky stimulus recognition as well as perception.

It is noticeable that the peak coordinate of the identified cluster in postcentral gyrus was far from the conventional finger representation in S1. For example, in the previous finger somatotopy study (7 Tesla fMRI study; Martuzzi et al., 2014), the coordinates of the center of mass for index finger was *x* = −51.6 ± 2.9, *y* = −23.1 ± 3.0, *z* = 58.4 ± 4.0 in postcentral gyrus. However, our searchlight analysis found the peak activation in postcentral gyrus at *x* = −64, *y* = −8, *z* = 14. This peak activation was rather closely located to S2, indicating the involvement of S2 activity in tactile stickiness information processing. This observation suggests that the cluster identified using the MVPA might contain specific spatial patterns of neural activities related to discrimination between different stickiness intensities, rather than mere detection of incoming tactile stimulation. Moreover, this is in line with the hierarchical view of human somatosensory networks: S1 is a main sensory receptive area for the sensation of touch and distributes somatic information to adjacent areas (e.g., S2, PPC) for higher level processing (Iwamura, 1998; Bodegard et al., 2001).

The second searchlight analysis, decoding the 5% vs. sham stimuli, sought brain regions in which the voxel response patterns of the Supra-threshold and Sham groups were distinctly present. Significant clusters were found in bilateral PPC, contralateral MTG, insula, and SMA, and ipsilateral thalamus, ITG, and ACC. These identified regions can be divided into two subgroups: Sensorimotor brain network including PPC, MTG, SMA, and thalamus (Vahdat et al., 2011) and salience brain network including insula, ITG, and ACC (Ham et al., 2013). Involvement of sensorimotor network with somatosensory perception is well known and previous neuroimaging studies have demonstrated modulation of functional connectivity in this network with tactile perception (Carey et al., 2011; Bannister et al., 2015). For instance, an fMRI study with stroke patients observed stronger functional correlations between the regions in the sensorimotor network after clinical improvement in touch discrimination (Bannister et al., 2015). In addition, our results revealed an involvement of salience network in decoding the Supra-threshold and Sham groups. Salience network responds to behaviorally salient events and mainly consisted of ACC and insula (Ham et al., 2013). The significant activations in salience network may be attributed to the obvious perceptual difference in two material properties. During the experiment, participants were asked to perceive stimuli with a focus on the tactile stickiness intensity. Since participants were paying attention to the stickiness intensity when the sham stimulus was presented, they might perceive an unexpected material difference as well as a stickiness intensity difference. As the sham stimulus was made of a different material (acryl) from other silicone sticky stimuli, it could be perceived as a salient stimulus. Therefore, it is speculated that the perception of different materials together with stickiness intensities might be related to distinguishable neural activity patterns in brain regions of salience network in response to the sticky silicone stimulus from those in response to the non-sticky acrylic stimulus.

The third searchlight analysis, decoding the Infra-threshold and Sham groups, did not identify any significant cluster. It is plausible in some sense, because this is consistent with our behavioral results (note that both 30% and sham stimuli were perceived as non-sticky). However, based on the fact that these two stimuli were made of two different materials, one may argue that this decoding analysis should activate salience network similar to the second searchlight analysis above. One putative explanation could be that the perceptual difference due to the distinct material properties was not recognized as salient because participants paid more attention to the stickiness intensity during the experiment. A further investigation is needed to verify this speculation.

While the first three searchlight analyses performed binary classification, the fourth analysis discriminated 3 different stickiness intensities (i.e., decoding the 5, 7, and 30% stimuli representing two Supra-threshold subgroups and Infra-threshold group). This analysis enabled us to explore neural activity pattern differences according to the variation of perceptual intensities. As a result, we found that multivoxel patterns in putamen varied with perceptual intensities of tactile stickiness. Previous studies have reported that putamen encoded a fine-grained aspect of somatotopy (Chudler and Dong, 1995; Bingel et al., 2004). Chudler and Dong showed that neurons in putamen increased their responses as tactile stimulus intensity was increased, arguing that tactile intensity information may be represented in the putamen (Chudler and Dong, 1995). Moreover, putamen area, together with thalamus identified from the first searchlight analysis, has been known as substantial parts of the basal ganglia–thalamocortical loop. Several previous studies demonstrated that this loop is associated with the somatosensory information processing (Kaji, 2001; Peller et al., 2006). In addition to these neurophysiological findings, our results lend support to the idea that putamen may play an important role in perceiving differential intensities of tactile stickiness.

### Comparison between uni- and multi-variate analysis results

In the previous and the current studies, we applied two different analytic approaches to the same fMRI dataset to seek neural activations implicated in tactile stickiness perception. It is thus worthwhile to compare the results obtained by uni- and multi-variate analyses. Generally, identified significant clusters from both approaches were distributed over somatosensory (postcentral gyrus and PPC), subcortical (basal ganglia and thalamus), and insula areas (insula and adjacent areas). However, there are several differences which are worthy of remark.

We could not find clear consistency of identified brain regions between the uni- and multi-variate analyses. For example, GLM analysis identified postcentral gyrus activations from Supra-threshold and Sham group contrast, while searchlight MVPA identified this area from the decoding of Supra- and Infra-threshold groups. This inconsistency might be explained by the difference of contrasting stimuli. In GLM analysis, we contrasted two perceptual groups with all stickiness intensities belonging to each group. On the other hand, the searchlight MVPA discriminated two perceptual groups using representative stickiness intensity only. The reason why we selected one representative intensity for each perceptual group was to match the number of samples for each group. This was an inevitable choice to eliminate the influence of having imbalanced groups for classification analysis (Pereira et al., 2009). For example, for the Supra-threshold and Sham group contrast, GLM analysis contrasted 5, 6, 7% against sham sticky stimuli, while searchlight MVPA decoded stickiness intensities between 5% and sham stimuli. In this case, unlike the GLM results, MVPA results could not imply the stickiness information of 6 and 7% stimuli. Hence, we assume that the difference of contrasting stimuli might be one of the reasons for the inconsistent results between uni- and multi-variate analyses.

For the purpose of a direct comparison between uni- and multi-variate results, we additionally performed three univariate analyses corresponding to the multivariate analyses: (1) 5% > 30%, (2) 5% > sham, and (3) 30% > sham. However, we could not identify a significant cluster from any of the three uni-variate analyses (*p* < 0.001 uncorrected, cluster size > 10). The absence of S1 activation was rather unexpected, because the low-level cues between Supra-threshold and sham stimuli were substantially different. There may be some possibilities related to this observation such that multi-voxel patterns would be able to distinguish different stickiness intensities while overall activation levels across a cluster of voxels were similar between stimuli (Davis et al., 2014) or that the number of trials for each contrast was too small to show consistent BOLD signal level differences.

In addition, significant activation was found in DLPFC by contrasting the Supra-threshold against Sham groups in the uni-variate analysis. However, multi-variate analysis did not find any significant activation in DLPFC. Since the activation in DLFPC has been associated with high-level cognitive processes such as decision making and emotional processing (for review, see Tanji and Hoshi, 2008), we had speculated that the DLPFC activation might reflect the emotional aspects (i.e., aversive states) aroused by sticky stimuli. However, our multi-variate results did not provide additional supporting evidence. Hence, our speculation here may need further justification with future work.

### Categorical representations of stickiness intensities

Using a dimensionality reduction method, the spatial organizations of the neural activity patterns were visualized in a 2-dimensional space. A follow-up categorization analysis using *k*-means clustering methods examined how brain activities in response to stickiness intensities could be grouped together. This allowed us to make a comparison of neural and behavioral representations of stickiness perception. The constructed low-dimensional neural representations exhibited clear grouping tendencies corresponding to sticky and non-sticky stimuli. It is remarkable that 5, 6% and 10, 30% sham stimuli were clustered into different groups in most cases (five of six cases; for detailed information, see Figure 3). On the other hand, we did not observe a consistent clustering pattern for 7, 8% stimuli across the multivoxel clusters. Interestingly, this neural categorization pattern was in line with the perceptual grouping pattern obtained by our psychophysical experiments. Absolute threshold for stickiness perception experiment revealed that the boundary catalyst ratio between perceptually sticky and non-sticky stimuli was 7.47 ± 1.3%. Hence, our results suggest that distributed activity patterns of fMRI signals may reflect the perceptual categorization of sticky sensations.

We applied *k*-means clustering methods to the PCA visualization derived from voxel response patterns as well as behavioral magnitude estimations in a 2-dimensional space. It showed how similarly neural and behavioral responses of stickiness intensities were grouped together and allowed a direct comparison between categorization patterns of neural and behavioral responses (Figure 3 and Figure 4). We observed one perfectly matched grouping pattern: When *k* = 2, behavioral responses to 5 and 6% stimuli were differently categorized from the remaining stimulus intensities and the same neural grouping pattern was found within the cluster identified the 5 vs. 7 vs. 30% stimuli decoding analysis. There were some discrepancies in other cases, but we could still observe substantial accordance between neural and behavioral categorization patterns. In particular, discrepancies were mainly found in 7, 8% stimuli, whereas 5, 6, 10, 30% stimuli were consistently categorized. Thus, one might speculate that a certain catalyst ratio between 6 and 10% presumably leads to different neural patterns in the human brain, which might play a role as a neural basis for tactile stickiness perception.

In this study, we investigated neural grouping patterns in the 2-dimensional space constructed by PCA. This linear dimensionality reduction method successfully revealed that neural categorization was consistent with the perceptual grouping pattern, but did not consider any potential non-linear characteristics. Therefore, it is worthwhile to explicitly model the underlying non-linear representations of fMRI signals using non-linear dimensional reduction methods such as Isomap (Tenenbaum et al., 2000) and Laplacian Embeddings (Belkin and Niyogi, 2003). Non-linear structures hidden in high-dimensional fMRI data will be explored in future work.

## Conclusion

This study investigated how neural activity patterns vary depending on the stickiness perception using a multi-variate approach. Searchlight MVPA showed that voxel response patterns in the human brain were distinguishable between sticky and non-sticky stimuli perception. Furthermore, the follow-up categorization analyses revealed that neural grouping patterns were consistent with the perceptual stickiness categorization pattern obtained by the psychophysical experiments. Therefore, our results indicate that distributed neural activity patterns in the human brain reflect tactile stickiness perception.

## Author contributions

JK participated in all aspect of the work, analyzed the data and wrote the manuscript. JY collected and analyzed the data and edited the manuscript. JR took part in the experiment as well as in the fMRI data acquisition. JP directed literature review and suggested a perspective on this study. SC provided theoretical bases. SK oversaw the study and managed every part of research.

### Conflict of interest statement

The authors declare that the research was conducted in the absence of any commercial or financial relationships that could be construed as a potential conflict of interest.
